# Comprehensive Shotgun Metagenomic Profiling of Antibiotic Resistance Genes in Sheep and Goat Farming Environments

**DOI:** 10.3390/antibiotics15030277

**Published:** 2026-03-09

**Authors:** Sara Gomes-Gonçalves, Jaqueline T. Bento, Guilherme Moreira, Joana Mourão, Rita Cruz, Fernando Esteves, Alexandra Lameira Baptista, Maria Aires Pereira, Pedro Caseiro, Pedro Carreira, Luís Figueira, João R. Mesquita

**Affiliations:** 1 School of Medicine and Biomedical Sciences (ICBAS), Universidade do Porto (UP), 4050-313 Porto, Portugal; 2 National Food Institute, Technical University of Denmark, Kongens Lyngby, 2800 Copenhagen, Denmark; 3 Instituto Politécnico de Viseu, Escola Superior Agrária de Viseu, Campus Politécnico, 3504-510 Viseu, Portugal; 4 Epidemiology Research Unit (EPIUnit), Instituto de Saúde Pública da Universidade do Porto, 4050-091 Porto, Portugal; 5 Laboratório para a Investigação Integrativa e Translacional em Saúde Populacional (ITR), 4050-600 Porto, Portugal; 6 CERNAS-IPV Research Centre, Instituto Politécnico de Viseu, Campus Politécnico, Repeses, 3504-510 Viseu, Portugal; 7 Universidade de Trás-os-Montes e Alto Douro, Quinta de Prados, 5000-801 Vila Real, Portugal; 8 Global Health and Tropical Medicine, GHTM, Associate Laboratory in Translation and Innovation Towards Global Health, LA-REAL, Instituto de Higiene e Medicina Tropical, IHMT, Universidade NOVA de Lisboa, UNL, Rua da Junqueira 100, 1349-008 Lisboa, Portugal; 9 Diogo Themudo-Sociedade Unipessoal Lda, Lugar Fontanheiras, 3520-112 Nelas, Portugal; 10 Arricom, Clínica Veterinária, Lda. Avenida General Humberto Delgado, Lote 4, Pá Ribeira, R/Chão, 2040-328 Rio Maior, Portugal; 11 Instituto Politécnico de Castelo Branco (IPCB), Av. Pedro Álvares Cabral 12, 6000-084 Castelo Branco, Portugal; 12 LAQV, REQUIMTE, Department of Chemistry and Biochemistry, Faculty of Sciences and Technology, University NOVA de Lisboa, 2829-516 Caparica, Portugal

**Keywords:** sheep, goat, Portugal, antimicrobial resistance, metagenomic

## Abstract

Background: Antimicrobial resistance (AMR) is a growing global health concern, driven in part by antibiotic use in animal production systems. Despite its relevance, the microbiome and resistome of small ruminant farm environments remain largely underexplored. Methods: In this study, shotgun metagenomics was applied to environmental samples from 46 sheep, goat and mixed-species farms across 14 municipalities in central Portugal. Results: Microbial profiling revealed a well-preserved microbiome with Pseudomonadota, Actinomycetota, Bacteroidota and Bacillota (*syn.* Proteobacteria, Actinobacteria, Bacteroidetes and Firmicutes respectively) as the most dominant phylum across different farm types. Regarding AMR, a total of 706 unique antimicrobial resistance genes (ARGs), covering 15 antibiotic classes, were detected. Tetracycline, aminoglycoside and macrolide resistance genes dominated across all samples, forming a conserved core resistome. While overall resistome profiles were broadly similar among farm types, significant differences were observed in specific ARG classes, such as pleuromutilin and fosfomycin. Conclusions: These findings highlight small ruminant farm environments as potential reservoirs of clinically relevant ARGs, including WHO highest priority critically important antimicrobial (HPCIA) resistance genes for macrolides (*mph(c)*, *erm(f)*, *erm(b)*) and fluoroquinolones (*qnrD1*), as well as critically important antimicrobial (CIA) resistance genes for glycopeptides (*vanR-SC*, *vanR-O*) and aminoglycosides (*str*, *aadA*), supporting the need to incorporate these environments into surveillance strategies.

## 1. Introduction

Antimicrobial resistance (AMR) poses a major global public health threat [1,2], with projections indicating 10 million AMR-attributable deaths per year by 2050 and worldwide economic losses exceeding $100 billion annually [3,4]. The emergence and spread of AMR are primarily driven by the misuse and overuse of antimicrobials in both human clinical settings, veterinary medicine, and agriculture [5]. The World Health Organization recognizes the animal production sector as a critical node in the One Health network for AMR mitigation because food animals constitute large reservoirs of bacteria carrying antibiotic resistance genes (ARGs) that can disseminate to humans via direct contact, environmental release, or the food chain [6,7,8].

Intensive livestock operations have historically received the greatest scrutiny in terms of AMR research, particularly in swine and poultry systems. However, extensive systems including sheep and goat farming also apply antibiotics therapeutically and prophylactically, thereby sustaining selective pressure that enriches the selection of ARGs-carrying bacteria [3,9,10]. While small ruminant systems have received less research attention compared to intensive operations, both sectors represent significant contributors to environmental AMR reservoirs, particularly in Mediterranean regions where extensive grazing predominates [11,12,13,14].

Extensive sheep and goat farming constitutes a central component of the Portuguese agricultural landscape, shaped by long-standing traditions, land availability and regional socioeconomic constraints [15]. Small ruminant production is predominantly carried out on family-operated farms with relatively small herds, reflecting the extensive nature of these systems and their close integration with natural grazing resources [16]. National statistics indicate that sheep and goat farming remain particularly relevant in less favored regions such as Alentejo and Beira Interior, where extensive areas of low agricultural suitability favor free-grazing systems and limit alternative agricultural activities [17]. In 2024, Portugal maintained approximately 2.1 million sheep and 0.3 million goats, with a substantial proportion of this population concentrated in these less favored regions, underscoring the structural importance of extensive small-ruminant production in marginal territories [17]. In these regions, extensive systems support a substantial proportion of the national small-ruminant population and play a key role in sustaining rural livelihoods and employment [15,18].

According to the European Surveillance of Veterinary Antimicrobial Consumption (ESVAC) data, Portugal reported significant veterinary antimicrobial sales in 2022, with tetracyclines (25.7%) and penicillins (17.6%) comprising the dominant antibiotic classes used in livestock production. Despite modest flock sizes, antimicrobial usage in sheep and goats is estimated at approximately 13.9 tons in 2022, representing 16.9% of Portugal’s total veterinary antibiotic consumption based on population correction unit (PCU) data [19,20,21]. These antibiotic classes are critically important for human medicine and consistent with global resistome surveys where they dominate environmental ARG profiles [22]. However, comprehensive metagenomic data on ARG diversity in extensive small ruminant systems are sparse in Portugal.

Existing studies, including a previous qPCR-based survey by our group [23] and culture-based investigations, have been limited to targeted detection approaches. While our qPCR approach detected *β*-lactam and tetracycline ARGs in 83% of environmental samples from 65 farms, this targeted approach captured only a small number of predefined genes. Comparable international research likewise reveals evidence vacuum [10]. Only 7 of 147 metagenomic livestock investigations published since 2018 included small ruminant matrices, and none combined multi-matrix sampling with high-resolution shotgun sequencing. Consequently, the full extent of resistome diversity, the taxonomic distribution of ARGs, and the broader microbial community structure remain uncharacterized. Shotgun metagenomic sequencing simultaneously profiles taxonomic composition, ARG diversity, and mobile genetic elements, enabling robust risk assessments of potential horizontal gene transfer events. This unbiased approach is indispensable for detecting novel or emerging resistance genes and assessing how resistance emerges and moves across ecosystems involving animals, humans, and the environment [24]. Here, we address some of these gaps by providing the first comprehensive, untargeted profile of the resistome and microbiome in Portuguese small ruminant farm environments.

This study characterizes the environmental resistome of sheep and goat farms in Central Portugal using Illumina shotgun metagenomics. By detecting ARGs in a key small ruminant production region, the findings contribute to assessing farming-related risks and inform One Health stewardship strategies. Ultimately, these insights advance targeted surveillance and mitigation efforts, supporting policy development for extensive small ruminant systems.

## 2. Results

### 2.1. Taxonomic Composition of Microbial Communities Across Farm Types

**Figure 1:**
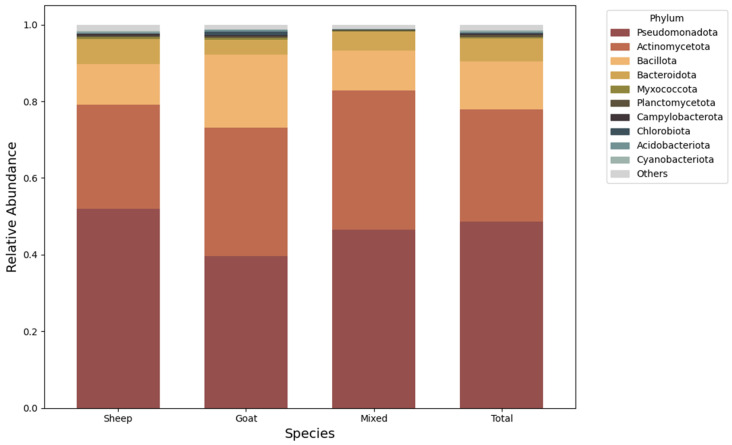
Relative abundance of bacterial phyla across species farm types. The stacked bar chart illustrates the taxonomic composition of microbial communities at the phylum level for sheep, goats, and mixed farm systems, with a weighted average provided in the “Total” column.

Across all sequenced samples, an average of 80% of reads were retained after quality filtering. Subsequent taxonomic filtering with Kraken2 removed a mean of 41.7% of post quality trim reads per sample (range: 30.6–63.8%), excluding non-target sequences prior to downstream analyses. Diversity and abundance of bacterial communities at the phylum level were assessed by farm type (Figure 1). Across all farm types, microbial communities were consistently dominated by Pseudomonadota, Actinomycetota, Bacillota, and Bacteroidota, which together accounted for most of the relative abundance in sheep, goat and mixed farms. Additional phyla, including Myxococcota, Planctomycetota, Campylobacterota, Chlorobiota, Acidobacteriota and Cyanobacteriota, were present at low relative abundances and showed no marked differences between groups. Overall, phylum-level community profiles were similar across all farms, with no statistically significant differences observed (*p* > 0.05).

### 2.2. Distribution and Abundance of Antibiotic Resistance Genes Across Farm Types

**Figure 2:**
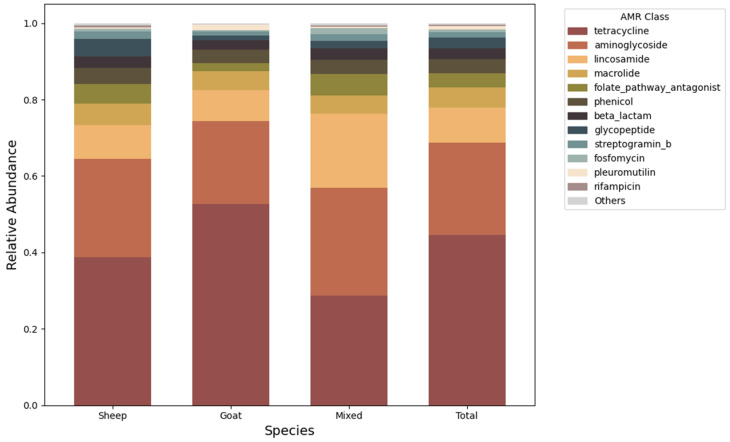
Relative abundance of ARG classes across species farm types. The stacked bar chart illustrates the diversity of ARG classes for sheep, goats, and mixed farm systems, with a weighted average provided in the “Total” column.

Shotgun metagenomic sequencing of composite environmental samples collected from sheep, goat, and mixed-species farms identified a total of 706 ARGs spanning 15 resistance classes. In terms of gene diversity, the most represented classes were beta-lactams (*n* = 163 unique genes), aminoglycosides (*n* = 143), and tetracyclines (*n* = 113). Considering gene abundance rather than diversity, tetracycline (44.65%) and aminoglycoside (24.07%) resistance genes were the most consistently dominant, followed by lincosamides (9.14%) and macrolides (5.27%) (Figure 2). Together, these four classes accounted for more than 70% of the total ARG relative abundance observed across all samples.

To evaluate whether resistome composition varied by farm type, ARG class profiles were compared across sheep, goat, and mixed-species farms. Overall, ARG class compositions were similar among the three farm types. Although minor variations were observed, pleuromutilin resistance genes were significantly more abundant in goat farms (ANOVA, *p* = 0.0307), and fosfomycin resistance genes were enriched in mixed-species farms (ANOVA, *p* = 0.0206). However, PERMANOVA indicated no significant differences in overall resistome composition across farm types (*p* > 0.05).

### 2.3. Correlation Network of Microbial Genera and Antibiotic Resistance Genes

**Figure 3:**
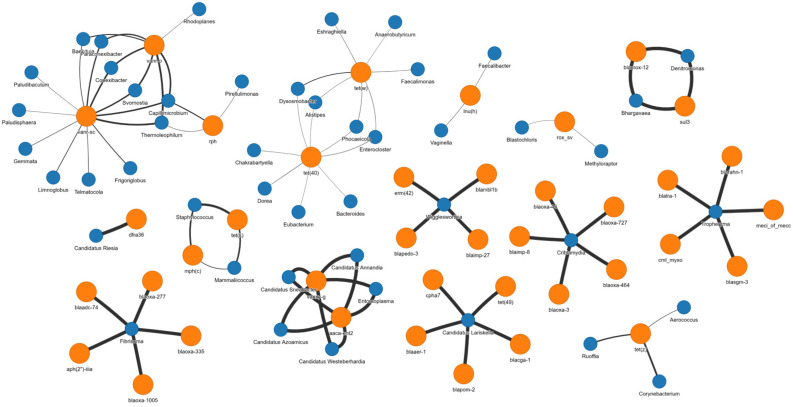
Correlation network showing significant positive associations between bacterial genera and antimicrobial resistance genes (ARGs) across samples. Blue nodes represent bacterial genera, while orange nodes represent ARGs. Edges represent significant positive Spearman correlations retained after filtering, with edge thickness reflecting the strength of the associations and higher statistical significance.

The correlation network analysis (Figure 3) revealed abundance-based associations between bacterial genera and ARGs across the pooled sample set. In this network, nodes represent genera or ARGs retained after correlation filtering, and edges represent significant positive Spearman correlations across samples. These associations indicate that samples with higher abundance of a given genus also tended to contain higher abundance of the associated ARG, or conversely, that the abundance of a given genus was positively associated with the abundance of a given ARG across all samples. However, it is important to note that is not possible to conclude, based on this analysis alone, that any genus carries or harbours a given ARG. Overall, many of the retained associations involved environmental and commensal taxa, consistent with the farm environmental context. Several ARGs conferring resistance to critically and highly prioritized critically important antibiotics (CIA and HPCIA) were primarily associated with environmental bacteria rather than genera more commonly linked to clinical pathogens.

Within this network, *Fibrisoma*, an environmental bacterium, showed positive associations with several *β*-lactamases, including *bla*_OXA-277_, *bla*_OXA-335_, *bla*_OXA-1005_, *bla*_ADC-74_, as well as the aminoglycoside resistance gene *aph(2″)-IIIa*. *Wigglesworthia*, an insect bacterial endosymbiont, was positively associated with the carbapenem resistance gene *bla*_IMP-27_ and the macrolide resistance *erm(42)*. The glycopeptide resistance gene *vanR-sc* formed one of the largest hubs in the network, showing multiple associations with environmental genera including *Baekduia*, *Svornostia*, *Conexibacter*, *Paraconexibacter*, *Capillimicrobium*, *Thermoleophilum*, and Planctomycetota members (*Gemmata*, *Frigoriglobus*, *Limnoglobus*, *Telmatocola*, *Paludisphaera*), whereas *vanR-o* showed fewer associations, mainly with *Rhodoplanes* and related genera. *Criblamydia* was also associated with several resistance genes, including the *β*-lactamases *bla*_OXA-3_, *bla*_OXA-46_, *bla*_OXA-464_, *bla*_OXA-727_, as well as the carbapenem resistance gene *bla*_IMP-8_.

In contrast, ARGs associated with genera that include pathogenic or opportunistic bacteria formed smaller and more network structures. *Tropheryma* appeared in a relatively isolated cluster with strong positive associations with several *β*-lactamases *(bla*_LRA-1_, *bla*_RAHN-1_, *bla*_SGM-3_). Opportunistic pathogens formed a separate cluster centered on tetracycline resistance genes. Within this cluster, *Staphylococcus* and *Mammaliicoccus* were associated with the tetracycline resistance gene *tet*(*k*), as well as with the macrolide resistance gene *mph*(*c*). *Corynebacterium*, *Aerococcus*, and *Ruoffia* were associated with *tet*(*z).* A distinct gut microbiota-associated cluster also emerged, centered on *tet*(40) and *tet*(*w),* involving *Enterocloster*, *Dorea*, *Chakrabartyella*, *Bacteroides*, *Phocaeicola*, *Dysosmobacter*, *Simiaoa*, and *Eshraghiella*. Overall, the network suggests that ARG occurrence in this system co-varied predominantly with commensal and environmental taxa, whereas associations involving genera that include pathogenic or opportunistic human-associated bacteria were less prominent.

## 3. Discussion

Livestock and their associated environments are recognized reservoirs of ARGs [25,26]. However, research on livestock-associated resistomes has predominantly focused on cattle and swine production systems [27,28,29,30,31], leaving small ruminant farms comparatively underexplored. To address this gap, the present study characterized the structure and variability of bacterial communities and ARG composition in environmental matrices from sheep, goat and mixed-species farms using metagenomic sequencing.

Microbial community profiling revealed that the most abundant phyla across all three farm types were Pseudomonadota, Actinomycetota, Bacteroidota and Bacillota. Research in other ruminant farm environments, such as cattle, has similarly reported the prevalence of these phyla across diverse sample types, including soil, manure and effluents [27,28,29]. Although minor differences in community evenness were observed among farm types, the relative stability of the dominant phyla suggests broad compositional similarity at high taxonomic resolution. This pattern is consistent with shared ecological pressures across farms, such as comparable feeding regimes and regional husbandry practices. In the central region of Portugal, autochthonous sheep and goat breeds largely rely on natural pastures, shrub vegetation and cereal stubble, with limited use of sown pastures [18]. In addition, regional husbandry practices, particularly transhumance, have been historically and broadly practiced by sheep and goat herders in the region [32].

Analysis of the farm resistomes revealed that tetracycline, aminoglycoside and macrolide resistance genes were the most prevalent ARG classes across all farms. These classes have been consistently reported as dominant in agricultural soils and livestock-associated microbiomes in previous studies [3,30,31]. Their uniform prevalence across farm types suggests a broadly shared environmental resistome in small ruminant systems. Although this pattern may be influenced by antimicrobial use in veterinary settings [33], the lack of detailed antibiotic consumption data from the studied farms prevents a direct link from being established. It is also important to note that these ARG classes are globally widespread and may persist in the environment independently of the antimicrobial consumptions [34,35,36]. Interestingly, ARGs conferring resistance to β-lactams showed the highest diversity in farm environments, although their relative abundance remained low. This pattern may be explained by two main factors. First, the diversity of β-lactamase genes is higher than that of ARGs associated with other antibiotic classes [37]. Second, β-lactam antibiotics are chemically unstable in the environment [38]. Compared with tetracyclines and macrolides, β-lactams degrade more rapidly [39], which may contribute to the reduced selective pressure in these settings.

The correlation network analysis provided important insights into ARG distribution patterns within farm environments. Most ARGs, including those associated with resistance to critically important antibiotics such as carbapenems, cephalosporins, and glycopeptides, were predominantly linked to environmental and commensal bacterial genera rather than to genera more commonly associated with pathogenic bacteria. This is consistent with previous work showing that ARGs in agricultural waste environments are commonly associated with environmental and non-clinical bacterial communities [40]. The predominance of environmental and commensal associations is noteworthy from a surveillance perspective. However, it is important to emphasize that ARG occurrence and correlation with particular genera do not by themselves demonstrate gene carriage, transfer risk, or clinical relevance. No pathogen isolation was performed in this study, and no gene expression data were generated. Therefore, the presence of HPCIA-associated genes such as mph(c), erm(b), and qnrD1 should be interpreted within an environmental surveillance framework rather than as direct evidence of transmissible or clinically actionable resistance. While horizontal gene transfer mediated by mobile genetic elements remains a theoretical concern in mixed microbial communities [41], the mobility context of the detected ARGs was not assessed in this study, and any inference regarding ARG dissemination potential remains speculative in the absence of evidence linking ARGs to plasmids, integrons, insertion sequences, or transposons.

From a public health perspective, the widespread presence of ARGs in farm-associated environments warrants attention, as such environments can contribute to the broader environmental reservoir of antimicrobial resistance affecting both veterinary and human health [42]. However, these findings should be interpreted as indicative of resistome composition in these underexplored agricultural settings, rather than as evidence of imminent public health risk. In fact, the present study did not assess ARG mobility, host association or exposure pathways, which are critical factors for evaluating public health risk. Accordingly, the results are best positioned as a foundation for future investigations that integrate mobility analysis, functional assays, and epidemiological linkage to more fully characterize the risk posed by farm-associated resistomes.

The application of metagenomic sequencing enabled a more comprehensive assessment of ARG diversity in environmental samples compared with targeted approaches such as qPCR, which was previously applied by our group to the same farm types [23]. In that study, detection rates for tetracycline, β-lactam and macrolide resistance genes were 22%, 39.2% and 17%, respectively, calculated as the proportion of farms in which each ARG class was detected. In contrast, metagenomic analysis in the present study identified these same ARG classes in nearly all farms, with detection rates of 100%, 98% and 95%, respectively. At the individual gene level, most genes targeted by qPCR, including erm(b), tet(a), tet(c), tet(m), tet(w), sul1, and sul2, were also detected in the metagenomic dataset. However, the clinically important ESBL genes blaCTX-M and blaTEM, which were specifically targeted by qPCR primers in the previous study, were not identified in the present metagenomic analysis. These discrepancies likely reflect methodological differences between approaches. Shotgun metagenomics allows alignment of sequencing reads to a wide range of sequences in reference databases, whereas qPCR is restricted to specific primer-targeted regions and may fail to detect divergent genes, particularly in the presence of inhibitors or sequence variation at primer binding sites [43]. However, qPCR typically offers higher sensitivity for low abundance targets [44,45], which may account for the detection of blaCTX-M and blaTEM in the previous study despite their absence in the metagenomic dataset. Their absence in shotgun sequencing data may reflect concentrations below detection limits rather than true absence. Nevertheless, the overall resistome structure observed here indicates dominance of ARGs associated with environmental bacterial communities rather than clinically relevant pathogens. Seasonal factors may also contribute to observed differences, as the previous study was conducted in spring and summer, while the present study was performed in autumn. Seasonal variation in ARG abundance has been reported in livestock farm environments, with higher ARG diversity observed in autumn and winter, reflecting the increased antibiotic use during these periods [46].

Despite the relevance of these findings, several limitations should be acknowledged. First, the use of a single composite environmental sample per farm, pooling water, bedding, pasture, and soil matrices, was a deliberate methodological choice driven by the scale of the study and the aim of capturing a representative farm-level resistome profile. However, this approach precludes the ability to attribute detected ARGs to specific environmental matrices and limits ecological inference regarding the distinct contributions of each microenvironment to the overall resistome. Future studies should analyze each matrix separately to better resolve ARG origins and improve the interpretation of ARG distribution across farm environments. Second, the absence of detailed records on antimicrobial administration limited the ability to directly link ARG prevalence to antibiotic use. Inclusion of farm-level treatment data in future work would strengthen causal inference. Third, this study focused on ARG detection and did not evaluate gene expression or functional activity. Approaches such as meta-transcriptomics or functional resistance assays would be required to confirm ARG expression and phenotypic relevance. Fourth, absolute bacterial abundance (total microbial load per sample) was not quantified, preventing assessment of the relationship between microbial load and ARG prevalence. Incorporating quantitative measurements in future studies would provide a more complete understanding of resistome dynamics in small ruminant farm environments. Finally, an important limitation concerns the mobility potential of the detected ARGs. Although several identified resistance genes are associated with WHO highest-priority critically important antimicrobials (HPCIAs), this study did not assess the genetic mobility context of these ARGs. Specifically, no analysis was performed to determine whether ARGs were linked to mobile genetic elements such as plasmids, integrons, or transposases, nor was host association with pathogenic bacteria investigated. As a result, statements regarding horizontal gene transfer potential and reservoir risk should be interpreted with caution, as the capacity for ARG dissemination beyond the farm environment remains speculative. Future studies should incorporate tools such as PlasmidFinder, MOB-suite, or others approaches used for the reconstruction and characterization of mobile genetic elements to better assess the mobilization potential of ARG and refine the public health risk assessment

## 4. Material and Methods

### 4.1. Sampling

**Figure 4:**
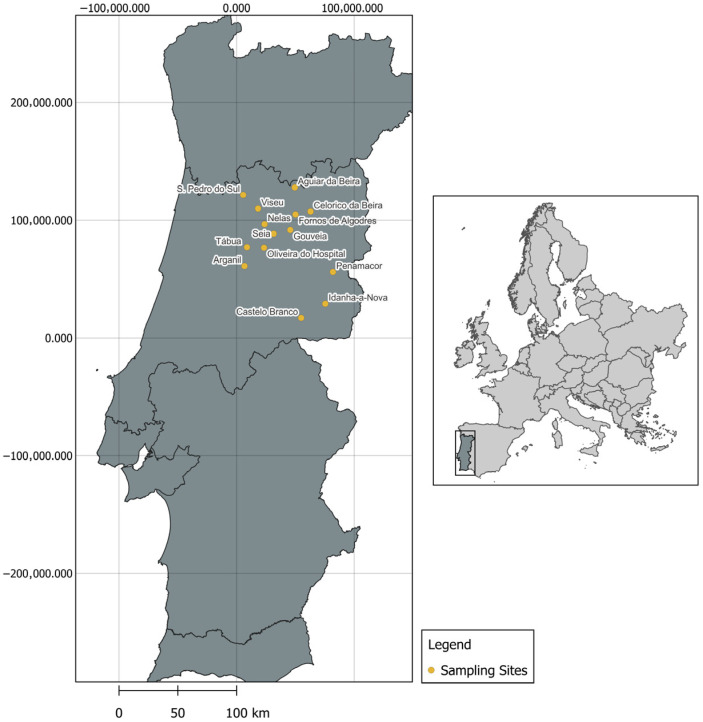
Geographical distribution of sampling sites.

A total of 46 farms were selected for the study, including 31 sheep, 11 goat, and four mixed sheep–goat operations (Table 1). Environmental samples were collected in September and October 2024 from farms included in a previous study [23], as well as from newly selected farms in Gouveia (*n* = 2), Celorico da Beira (*n* = 1), and Fornos de Algodres (*n* = 1). Geographical representation is shown in Figure 4.

From each farm, one composite environmental sample was collected, resulting in 46 samples for metagenomic analysis. The composite environmental samples were collected from four distinct microenvironments: (i) drinking water sources, (ii) animal bedding areas, (iii) grazing pastures, and (iv) soil from livestock housing areas. This composite sampling strategy was adopted to maximize farm-level resistome coverage within the logistical and financial constraints of a large-scale metagenomic study spanning 46 farms across multiple municipalities. By pooling matrices, the approach captures a broad representation of the farm resistome, which is consistent with the primary objective of characterizing resistome composition at the farm level rather than resolving matrix-specific ARG contributions.

For composite sample preparation, 500 mL of drinking water was collected from animal troughs or natural sources and immediately filtered through sterile 0.44 μm cellulose acetate membranes (Millipore, Burlington, MA, EUA). Solid samples consisting of 0.25 g each of bedding material, surface soil, and pasture material were collected using sterilized tools. The water filter was combined with three solid sample components (0.25 g each) to create a single composite environmental sample representing each farm’s resistome profile. This composite environmental sample was then used for DNA extraction (see Section 4.2 for further details). Sample IDs were assigned based on a numerical code and the type of animals present on each farm, using “O” for sheep, and “C” for goats. IDs containing both “O” and “C” correspond to farms where both species cohabit.

To prevent cross-contamination, sampling personnel wore disposable nitrile gloves, changed between each sample type and sampling point. Samples were immediately placed in sterile, pre-labeled bags and stored in insulated containers with ice packs. Upon arrival, samples were stored at −20 °C until further analysis.

### 4.2. DNA Extraction and Next-Generation Sequencing

Genomic DNA was extracted from each composite environmental sample, which consisted of 0.25 g of each solid sample type (bedding, pasture, and soil) combined with the water filter collected from each farm. Extractions were performed with the GRS Genomic DNA Kit–Soil^®^ (GRISP, Porto, Portugal) according to the manufacturer’s instructions. DNA yield was quantified by Qubit fluorometry (Thermo Fisher Scientific, Waltham, MA, USA). Extracted DNA was stored at −20 °C before shipment to Novogene UK (Cambridge, UK, CB4 0FW). Shotgun metagenomic library preparation was performed according to Illumina-compatible protocols by Novogene. Sequencing was performed on the NovaSeq X Plus platform, generating approximately 9 Gb of raw sequence data per sample.

### 4.3. Bioinformatic Analysis

Raw read quality control was performed using Fastp v0.23.2 [47] with the following parameters: quality trimming of bases with Phred scores < 15 (—qualified_quality_phred 15), removal of reads shorter than 50 bp after trimming (—length_required 50), and automatic adapter detection and removal for paired-end reads (—detect_adapter_for_pe). Read counts and quality metrics were recorded before and after filtering. Quality-filtered reads were processed through a two-step taxonomic classification workflow to remove host-derived sequences and profile microbial taxa. In the first step, reads were aligned against Ovis aries (GCF_016772045.2) and Capra hircus (GCF_001704415.2) reference genomes using BWA-MEM (v0.7.17) [48] with default parameters. Reads mapping to either host genome (primary alignments with MAPQ ≥ 30) were removed, and only unmapped reads were retained for further analysis. In the second step, the host-depleted reads were taxonomically classified with Kraken2 using a prebuilt RefSeq plusPF database, 7/14/2025 version [49], which includes representative Refseq genomes of archaea, bacteria, viral, plasmid, human, plant and fungus genomes, along with representative sequences from UniVec_Core [50]. A confidence threshold of 0.1 (—confidence 0.1) was applied to reduce ambiguous assignments. For downstream microbial profiling, only reads classified as bacteria or archaea were retained. Archaea were retained to preserve ecological completeness and capture their potential role as environmental reservoirs of antimicrobial resistance genes [51,52,53]. The resulting bacterial and archaeal classifications were refined using Bracken v2.8 [54] to improve genus- and species-level abundance estimates. Taxa with <20 estimated reads per sample were excluded to minimize the influence of low-confidence assignments and focus on robust, high-abundance genera. To characterize and quantify the diversity and abundance of ARGs, the previous cleaned reads were aligned to the PanRes database v1.0.1 [55] (https://zenodo.org/records/18606078 accessed in 17 January 2024), a comprehensive compilation of multiple ARGs databases, using ARGprofiler [55] (v2.0, https://github.com/genomicepidemiology/ARGprofiler accessed on 17 January 2024) with default settings. Gene-level abundance for each sample was quantified as the mean per-base coverage (sequencing depth) of each ARG. Overall mapping rates to the ARG database were recorded. The relative abundance of individual ARGs within a sample was determined by dividing the mean per-base coverage of each gene by the total ARG coverage across all genes, expressing each gene’s contribution as a proportion of the overall resistance gene signal. ARGs were subsequently categorized into resistance classes based on PanRes database annotations. Class-level abundances were calculated for each sample by summing the per-gene coverage values within each class and normalizing by the total ARG coverage, yielding relative abundances. ARG and taxonomic profiling results were integrated with sample metadata, specifically host species (sheep, goat, or mixed), to construct a unified data matrix. Data visualization and summary tables were generated using custom Python (v3.10.12) scripts available at https://github.com/GmoreiraVet/RUmiRes2 (accessed on 17 January 2024). ARG profiles were further characterized by aggregating the per-gene coverage values for each sample into a gene-by-sample matrix. To account for differences in scale across genes, this matrix was standardized using z-score normalization.

### 4.4. Statistical Analysis

Relative abundances of bacterial phyla were calculated from Bracken output files using the fraction of total reads assigned to each phylum per sample. Phyla with consistently low abundance (<1% across all samples) were collapsed into a single “Others” category to reduce visual noise and facilitate interpretation. For ARG analyses, gene depths were summed per sample and class and normalized to total ARG depth to obtain relative abundances. Differences in normalized ARG class abundance among host species were assessed using one-way analysis of variance (ANOVA) models, with each factor treated as a categorical predictor. Prior to ANOVA, model assumptions were evaluated by inspecting residual Q–Q plots to assess normality and residuals versus fitted value plots to assess homoscedasticity. Although relative abundance data may show minor deviations from normality, ANOVA is considered robust under balanced group designs.

To account for multiple testing across ARG classes, *p*-values obtained from the univariate ANOVA models were adjusted using the Benjamini–Hochberg false discovery rate (FDR) procedure, and statistical significance was determined based on FDR-adjusted *p* values. Overall differences in resistome composition among host species were further evaluated using permutational multivariate analysis of variance (PERMANOVA) based on Bray–Curtis dissimilarity with 999 permutations. All analyses were performed in Python using pandas (v2.3.3), SciPy (v1.15.2), statsmodels (v0.14.6), and scikit-bio (0.7.2).

### 4.5. Correlation Analysis

Correlations between genera and AMR genes were evaluated using genus-level abundance data obtained from Bracken and AMR gene abundance profiles generated from metagenomic sequencing data. Pairwise associations between genera and AMR genes across samples were evaluated using Spearman’s rank correlation coefficient (ρ). Only positive correlations above a predefined threshold (ρ > 0.85 and *p* < 0.05) were retained for downstream analysis. Network representations were built in Python using pandas for data handling, NetworkX (v3.4.2) for graph building, and matplotlib (v3.10.1) for visualization.

## 5. Conclusions

This study provides the first comprehensive metagenomic characterization of the microbiome and resistome in sheep, goat, and mixed-species farm environments in Portugal. Across all farm types, microbial communities were dominated by Pseudomonadota, Actinomycetota, Bacteroidota, and Bacillota, revealing a conserved core microbiome. The resistome was similarly stable, with tetracycline, aminoglycoside, and macrolide resistance genes consistently dominant across farms, highlighting a conserved environmental reservoir of ARGs in small ruminant systems.

From a public health perspective, the prevalence of ARGs associated with clinically relevant bacterial underscores the importance of including small ruminant farms in antimicrobial resistance surveillance frameworks. Future research should aim to determine ARG expression, quantify microbial loads, assess the contribution of antibiotic use, and investigate the specific sources and mobility of resistance genes within these environments to better inform mitigation strategies.

## Figures and Tables

**Table 1:** Geographic distribution of sheep, goat and mixed farms included in the study by municipality.

Municipality	Sheep Farms (*n*)	Goat Farms (*n*)	Mixed Farms * (*n*)
Aguiar da Beira	3	2	0
Arganil	0	1	0
Castelo Branco	1	0	0
Celorico da Beira	3	1	1
Fornos de Algodres	1	1	1
Gouveia	3	1	2
Idanha-a-Nova	1	0	0
Nelas	1	0	0
Oliveira do Hospital	2	0	0
Penamacor	7	2	0
S Pedro do Sul	5	2	0
Seia	2	0	0
Tabua	1	0	0
Viseu	1	1	0
Total	31	11	4

* Mixed farms represent farms where sheep and goat share the same environmental matrix (bedding, pasture, soil, and water).

## Data Availability

The original contributions presented in this study are included in the article. Further inquiries can be directed to the corresponding author.
